# Beyond handwashing: Water insecurity undermines COVID-19 response in developing areas

**DOI:** 10.7189/jogh.10.010355

**Published:** 2020-06

**Authors:** Justin Stoler, Wendy E Jepson, Amber Wutich

**Affiliations:** 1 Department of Geography, University of Miami, Coral Gables, Florida, USA; 2 Department of Public Health Sciences, Miller School of Medicine, Miami, Florida, USA; 3 Department of Geography, Texas A&M University, College Station, Texas, USA; 4 School of Human Evolution and Social Change, Arizona State University, Tempe, Arizona, USA

The global response to COVID-19 has invoked the familiar refrain “back to basics” with respect to basic control strategies such as quarantining and isolation, handwashing, and social distancing. The pandemic has already revealed stark structural challenges to the health care and governance systems of many high-income nations. As of March 2020, we have only seen a glimpse of how COVID-19 may affect low- and middle-income nations that have even fewer material resources. In this context – particularly in high-density urban areas that are potential epicenters of transmission – perhaps no other single factor will impede control strategies as the daily struggle experienced by billions of households globally: water insecurity.

Water, sanitation, and hygiene (WASH) inequities are long-recognized as important contributors to the global burden of disease that inhibit sustainable development [1]. Some media outlets have already described how the lack of water may limit handwashing, as scarce water is often prioritized for other tasks [2]. We are already seeing a glimpse of this in Navajo Nation, where COVID-19 cases have spiked in communities with limited water service [3]. In Mexico City residents have reportedly left their homes for relatives’ residences with a more reliable water supply, as increased urban water demand is stress-testing a precarious water system [4]. Inadequate water quantity poses additional challenges for maintaining clean environments and sanitizing physical surfaces where COVID-19 can survive. But the problems associated with water insecurity extend well beyond issues of quantity and hygiene.

Social distancing can cause significant disruptions in people’s access to their most basic necessity. One of the most underappreciated coping strategies for dealing with water insecurity is water sharing between households. The ubiquitous, but often invisible, practice of household water sharing occurs in a variety of socio-cultural settings [5], and may serve as a transmission pathway for many communicable diseases [6]. Although there is currently no evidence that COVID-19 can survive in drinking water [7], the act of water sharing involves practices that contradict advice of social distancing. Water sharing can facilitate COVID-19 transmission through close interpersonal interactions – often in other people’s living quarters – and physical contact with containers and taps. Even where social distancing is practiced in a water sharing arrangement, such as leaving out water for a neighbor, or allowing self-service from a private well, there is still overlap in physical activity spaces and interactions with potentially contaminated objects.

Water insecurity also complicates people’s ability to participate in social distancing if they have to fetch their own water. About a billion people globally collect their own water from sources outside of their home such as public standpipes, wells, or surface water bodies, and often at great distances. The process of fetching frequently involves queuing in close proximity (to avoid losing one’s place in line), and may be contingent upon water availability if water systems are rationed. Water fetching in groups contributes to building social capital among women and children, while offering a mechanism of protection from physical dangers like injuries, accidents, harassment, and assaults [8,9]. Thus, when social distancing measures are in place, vulnerable groups (especially women and girls) experience greater risks by having to fetch water alone.

These disruptions reinforce the experience of water insecurity, which comes in many forms beyond physical access, such as social exclusion and psychosocial effects [10,11]. Resource stress is a major trigger for interpersonal violence, and many households are already beginning to experience resource stresses related to job loss, food insecurity, medical bills, and other economic impacts of COVID-19 [12]. Recent research has shown that women in water-insecure households are more likely to suffer intimate partner violence [13], and this is likely to be intensified in the contexts of the COVID-19 pandemic and related economic downturn. Given that pandemic-related stigma is already emerging, we should expect and plan for such stigmas to be intensified among groups that are experiencing water insecurity, poverty, ethnic discrimination, and other forms of social exclusion [14].

Households in low- and middle-income countries are commonly dependent on multiple water sources for domestic needs [15]. This water is either completely or partially distributed through informal systems, such as water trucks, unlicensed and commercial vendors, and informal arrangements with neighbors [16,17]. These informal systems may feel pressure to temporarily cease operations in the name of social distancing and other societal demands of those providers. Unlike public services, informal water supply systems and their workforce are not obligated to operate these unregulated supply chains under emergency conditions.

**Figure:**
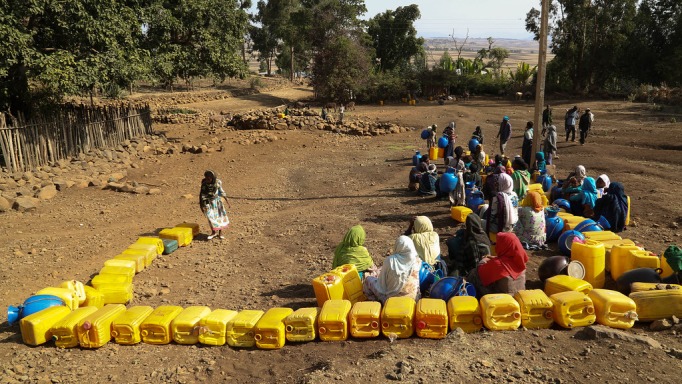
Photo: A borehole for people in Mankorkoria and Ansela Kebele, Ethopia, serves 400 households who are allowed a couple of jerrycans every other day. By Nahom Tesfaye, UNICEF Ethiopia, licensed under CC BY-NC-ND 2.0.

We also know that as supplies decrease or become intermittent and unreliable, households cope by storing water. Bottled water is one way to store water, as we have seen in high-income settings where panic buying has emptied stores of bottled water inventory and left water-insecure households with no water source [18]. In lower and middle-income countries, water storage technologies may amplify public health risks [19]. Improper storage practices and poor quality or inappropriate storage containers may increase microbial recontamination or promote breeding of insect vectors. While household water treatment systems can effectively reduce health risks from unsafe drinking water in acute emergencies [20], social distancing under conditions of a global pandemic requires creative strategies to provide requisite supplies and training for households and communities.

All of these issues are exacerbated by the human ecology of slums and informal settlements that are home to a billion people [21]. These living conditions typically feature limited access to basic WASH services amid high population density, low quality housing, and myriad risk factors that present comorbidities [22]. The combination of high density and low quality housing limits the effectiveness of isolation and quarantining because people are in close proximity with substandard barriers, particularly in parts of the world where many people share bedrooms, or where multiple households cohabitate in compound housing. There is often simply nowhere to go, and this may prompt behavior changes, such as sleeping outside, that expose people to environmental risk factors that reinforce the kinds of comorbidities associated with the most serious COVID-19 symptoms. Such environments are not only breeding grounds for infectious disease risk, but also for the social stigma that marginalizes the sick, prevents them from accessing care, and increases mortality [14].

## CONCLUSION

Under-resourced regions that already suffer economic losses from lack of adequate water supplies [23] are on the verge of an enormous additional burden from COVID-19. We can implement policies and practices with a greater probability of adherence, and ultimately save lives, by appreciating the complexity of how water insecurity interacts with COVID-19 control measures. We affirm the notion of getting “back to the basics” in order to contain and halt this pandemic. But once COVID-19 is under control and we have taken stock of the damage to global health systems, we hope the world will pivot back to basic services, such as close, reliable, safe, and secure water. It will cost us much more if we do not.
